# TIME – Targeted interdisciplinary model for evaluation and treatment of neuropsychiatric symptoms: protocol for an effectiveness-implementation cluster randomized hybrid trial

**DOI:** 10.1186/s12888-016-0944-0

**Published:** 2016-07-12

**Authors:** Bjørn Lichtwarck, Geir Selbaek, Øyvind Kirkevold, Anne Marie Mork Rokstad, Jūratė Šaltytė Benth, Janne Myhre, Solvor Nybakken, Sverre Bergh

**Affiliations:** Centre for Old Age Psychiatric Research, Innlandet Hospital Trust, Ottestad, Norway; Institute of Health and Society, Faculty of Medicine, University of Oslo, Oslo, Norway; Norwegian National Advisory Unit on Ageing and Health, Vestfold Hospital Trust, Vestfold, Norway; Departement of Health, Care and Nursing, Faculty of medicine NTNU, Norwegian University of Science and Technology, Gjøvik, Norway; Molde University College, Faculty of Health Sciences and Social Care, Molde, Norway; Institute of Clinical Medicine, Campus Ahus, University of Oslo, Oslo, Norway; HØKH, Research Centre, Akershus University Hospital, Lørenskog, Norway

**Keywords:** Dementia, Neuropsychiatric symptoms, Behavioural and psychological Symptoms of dementia (BPSD), Psychosocial interventions, Nursing home, Case conference

## Abstract

**Background:**

Nearly all persons with dementia will experience neuropsychiatric symptoms (NPS) during the course of their disease. Clinicians and researchers emphasize the need for an evidence-informed standardized approach to managing NPS that integrates pharmacological and nonpharmacological treatments for real-world implementation. The Targeted Interdisciplinary Model for Evaluation and Treatment of Neuropsychiatric Symptoms (TIME) represents such an approach and is a multicomponent intervention based on the theoretical framework of cognitive behavioural therapy.

**Methods/design:**

The trial is a 3-month cluster randomized trial conducted in 30 nursing homes including 168 participants with dementia and a high level of agitation. Each nursing home defined as a cluster will be randomized to receive either the TIME intervention (the intervention group) or a brief education-only intervention regarding dementia and NPS (the control group). TIME is a manual-based, multicomponent programme that includes a rigorous assessment, one or more case conferences and the treatment and evaluation of NPS. Patient-level measurements are taken at baseline (prior to randomization) and 8 and 12 weeks later. The primary outcome measure is the change in agitation, as defined by the Neuropsychiatric Inventory-Nursing Home Version, at 8 weeks from baseline. Secondary outcome measures include change in agitation at 12 weeks from baseline, and change from baseline at 8 and 12 weeks in other NPS, quality of life, and the use of psychotropic and analgesic medications. Mixed methods will be used to follow, measure and explore the implementation process and the effect of the intervention at the individual staff level and the organization level. Combining measurements of clinical effectiveness and implementation outcomes define this trial as an effectiveness-implementation hybrid trial.

**Discussion:**

Measuring the implementation and effect of complex interventions aimed at reducing NPS in nursing homes is challenging. In this study protocol, we describe a multicomponent program, TIME, and discuss how an effectiveness-implementation cluster randomized hybrid trial can meet these challenges.

**Trial registration:**

ClinicalTrials.gov identifier NCT02655003. Registered 6 January 2016.

## Background

In Norway, approximately 41,000 persons live in nursing homes, and more than 80 % of these have dementia [1, 2]. Nearly 70 % of persons with dementia in nursing homes exhibit clinically significant neuropsychiatric symptoms (NPS)—also labelled as behavioural and psychological symptoms of dementia (BPSD), such as psychosis, depression, anxiety, agitation and apathy [3]. NPS like agitation, including physical or verbal aggression and excessive motor activity cause patients to experience profound suffering and a reduced quality of life and caregivers to experience increased burden [4]. These symptoms represent great challenges in the care of nursing home patients and are predictors of referral to specialist health care and hospitalization [2, 5]. In a review article published in 2014, a multidisciplinary expert panel emphasized the need to develop comprehensive models for the assessment and treatment of these symptoms. Such models should enable the simple implementation of these recommendations in real-world settings [6].

A literature review by Livingstone et al. concluded that behavioural therapeutic techniques and psychoeducation aimed at altering the caregiver’s behaviour seemed to reduce NPS [7]. However, the findings regarding other types of treatment were inconclusive and inadequately documented. In a literature review that Testad et al. performed in 2014 on personalized interventions targeting NPS the authors noted increasing evidence that such interventions reduce NPS [8]. In a controlled trial, Cohen-Mansfield et al. showed that a systematic individual intervention based on a step-by-step algorithm significantly reduced agitation. Unfortunately, this study excluded patients exhibiting physical aggression, and the research team implemented the treatment measures in the wards [9]. Testad et al. conducted a randomized intervention trial in nursing homes in Norway and found that the systematic education and supervision of staff resulted in a reduced use of restraints although the level of agitated behaviour remained unchanged or increased slightly [10]. A systematic review by Reuther et al. including 432 studies of case conferences performed as interventions to address challenging behaviour concluded that four of seven studies in the analysis showed a reduction in the challenging behaviour of people with dementia [11]. The review highlighted the need for methodologically well-designed intervention studies. A disadvantage of many of these interventions is that they require a substantial amount of additional resources to nursing homes to be implemented successfully. A systematic review performed by Seitz et al. (2012) included 40 studies on various interventions aimed at reducing NPS. Sixteen studies showed positive results, but 75 % of them required a significant increase in the resources available to the nursing homes [12]. To our knowledge no trials have used principles from cognitive behavioural therapy (CBT) to structure care-delivery interventions to manage NPS in nursing homes.

### The development of the TIME intervention

The Targeted Interdisciplinary Model for Evaluation and Treatment of Neuropsychiatric Symptoms (TIME) was developed in nursing homes by the first author, BL. This model has been used in clinical practice in many nursing homes since it was first developed in 2007–2008. It requires minimal training, is manual based, and is easy to integrate into everyday clinical practice and care without major additional costs. The model is based on the theoretical frameworks of cognitive behavioural therapy (CBT) and person-centred care (PCC), which state that human behaviour is subject to the continuous influence of biological, social and psychological factors. Thus, the model integrates pharmacological and nonpharmacological treatments for real-world implementation. The primary purpose of the model is to allow an interdisciplinary team of staff and physicians to conduct a thorough assessment and critical systematic reflection in case conferences to achieve a mutual understanding of NPS and, thereby, implement customized actions based on this understanding. In 2010, The Centre for Old Age Psychiatric Research, Innlandet Hospital Trust conducted an open non-controlled trial in nine nursing homes over six months and included 30 persons with dementia and high levels of agitation. The results showed that patients’ agitation, mood symptoms and staff’s distress were significantly reduced. This study was published as an abstract in International Psychogeriatrics [13] and formed the basis for a revision of the TIME manual [14] and a web-accessible short film that can be used for training in the model [15]. In this abstract, the model was referred to as the Multidisciplinary Intervention Model for Challenging Behaviour in Nursing Home Patients with Dementia (MIND).

### Research aim and research questions

The primary purpose of this study is to improve the assessment and treatment of agitation in persons with dementia by examining the effect and implementation of the TIME intervention model. We formulated the following research questions: 1) Can an intervention utilizing TIME reduce agitation in persons with dementia in nursing homes? 2) Does TIME serve as a method of continuous learning and reflection? That is, can the model help develop and strengthen staff members’ confidence, mastery and competence at an individual level and at an organizational level? 3) What nursing home factors inhibit or promote the implementation of psychosocial intervention models such as TIME, and is implementation sustainable?

## Methods and design

### Study design

**Fig. 1:**
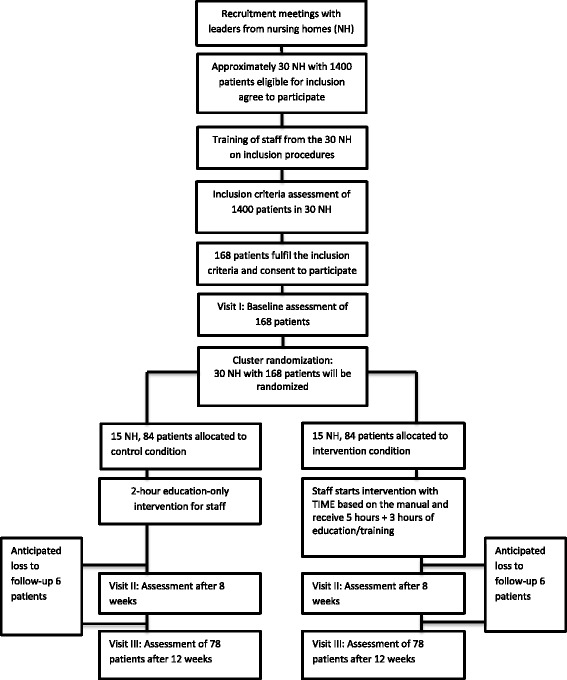
The TIME trial: Flowchart of the clusters and individuals throughout the phases of the trial

The first research question will be answered through a cluster randomized controlled trial with two parallel groups: Intervention Nursing Homes (INH) and Control Nursing Homes (CNH). Fig. 1 shows a flow chart of the clusters and individuals through the phases of the trial based on the power calculation. For the second and third research questions, we will utilise both quantitative and qualitative methods. Data will be gathered from the records of reflection meetings (case conferences) held during the trial, implementation checklists completed during the trial, four focus group interviews performed after the trial, and questionnaires administered before and after the trial. The trial is defined as an effectiveness-implementation cluster randomized hybrid trial because of the use of various types of data and the study design.

### Settings and target population

Municipalities located in the north, middle and south-eastern part of Norway will be contacted to participate in the trial. To ensure collaboration and implementation throughout the trial, we will arrange meetings with the health care leaders in the municipalities and the managers and physicians working in each nursing home. We will strive to recruit nursing homes located in both rural and urban areas of Norway and to obtain an equal distribution of large and small nursing homes to ensure a representative nursing home population. Nursing homes already using TIME as part of their clinical routines will not be invited to take part in the trial.

All patients in wards in participating nursing homes will be considered eligible for inclusion in the trial, and will be assessed to determine if they meet the inclusion criteria. Trained nursing home staff will perform the assessments. The research team will train these staff members on the inclusion procedure and the assessment of patients’ capacity to provide consent. The data obtained from screening individual eligible patients will not be recorded. Patients who fulfil the inclusion criteria and agree to participate will be included in the trial. For patients who lack the capacity to provide consent, written consent will be obtained from their next of kin.

The inclusion criteria for patients are as follows:

- NH patients with probable dementia, defined as a Clinical Dementia Rating scale (CDR) [16] score of one or higher.
- A moderate to high degree of agitation, defined by an NPI-NH agitation item score equal to or above six points.
- Long-term patients who have resided in the nursing home for a minimum of two weeks before inclusion.

The exclusion criterion for patients is a life expectancy of less than 4–6 weeks.

### Sample size calculation based on the primary outcome

The primary outcome of the trial is the change in the level of agitation from baseline at eight weeks, as measured by the agitation item of the Neuropsychiatric Inventory-Nursing Home Version (NPI-NH) [17, 18]. Power calculation was based on the following assumptions. A previous non-controlled pilot study of TIME showed that the intervention decreased the NPI-NH agitation item score by on average 2.8, with a standard deviation (SD) for change of 3.1 [13]. One can reasonably assume that the simple education-only intervention and baseline and follow-up assessments may have some effect on the control group. Therefore, we expect that the difference in the effect between the control and intervention groups will be lower. We have assumed a mean difference between the groups to be 1.5 as measured by NPI-NH agitation item, and that this difference will have a SD of 3.1. Based on this assumption, we calculate that 65 persons must be included in each group to observe a statistically significant difference with a power of 80 % and a significance level of 5 %. We assume an intra-cluster (nursing home) correlation coefficient (ICC) of 0.05. The ICC is assumed to be low because the persons included will be located in different departments and units in the nursing home (the cluster). Adjusting power calculations for cluster effect, we find that at least 78 persons have to be included in the intervention group and 78 in the control group for the effect to be statistically significant. That is, we need a total of 156 persons. Based on previous studies, we assume that the average size of the nursing homes is 46 patients and that each nursing home will recruit five patients, on average. In the pilot study, we found that approximately 12 % of patients had dementia and an NPI-NH agitation item score of six or higher, which is our main criterion for inclusion. Previous studies have shown that we can anticipate a 30 % loss to follow-up per year (due to, e.g., mortality, relocation, or withdrawal from the study)—that is, 7.5 % in three months. Thus, we will need a total of 168 people (84 persons in each group), indicating that we will need to screen approximately 1400 nursing home patients. Given approximately 46 patients on average per nursing home, we will need to recruit approximately 30 nursing homes. The nursing homes will be randomized after the baseline assessment to avoid bias. The recruitment of new patients to the study will therefore occur only through the recruitment and randomization of new nursing homes, as described above.

This study must perform cluster randomization, with the nursing home as the cluster, for two main reasons. The TIME intervention is a biopsychosocial intervention that involves the entire interdisciplinary team and staff in the wards of the participating nursing homes to optimize the treatment provided to a group of patients in the wards. In addition, the study runs the risk of transmitting all or parts of the intervention model to the control units or individual control patients at the same nursing home [19].

### Randomization

Nursing homes will be stratified by size into three blocks to assure approximately equal number of patients in the two trial arms. Block size will be fixed—block 1: 1–5 patients, block 2: 6–9 patients, and block 3: 10 or more patients. Then, nursing homes within each block will be randomly assigned to either the intervention group or the control group. A researcher will perform the randomization procedure independently of the project management team and the nursing homes. The project management team will provide the nursing homes with the randomization and allocation results immediately following this procedure. Specially trained project nurses who are not affiliated with the nursing homes will assess patients’ baseline characteristics before randomization. These assessors will also assess the effect of the intervention via telephone at weeks eight and 12 and will be blinded to the randomization result.

### Control and intervention phases of the study

#### Similar education and training for the staff in CNH and INH—CNH continue practice as usual

Three nurses in each unit in both the INH and CNH will be given a special responsibility in the trial. Before randomization, these nurses will complete a three-hour training on the procedure. Their main task will be to facilitate the interviews for the assessments at baseline, and after 8 and 12 weeks. These nurses will be selected by the leading ward registered nurse based on the following criteria: nurses who work on a nearly full-time basis, have shown interest in professional development and have gained legitimacy with the rest of the staff. Thus, these nurses can be selected among registered nurses, auxiliary nurses, nursing aides or members of other professional groups in the nursing homes.

After randomization, the staff in the INH and the CNH will be offered a two-hour lecture about dementia and NPS. This lecture represents the education-only intervention administered to the staff in the CNH. These staff members will then continue practice as usual for the patients throughout the remainder of the trial.

### Exclusive education and training of staff in the INH—intervention utilizing TIME in the INH

In addition to the two-hour lecture about dementia and NPS, the staff in the INH will complete three hours of lectures, training and roleplay related to TIME. The education and training team responsible for conducting the education and training sessions consists of the project management team (a physician with special competence in nursing home medicine and two specialist registered nurses in geriatrics) and four specialist registered nurses in old age psychiatry, all of whom are familiar with TIME. The lectures will be standardized according to the steps listed in the TIME manual.

The leading ward registered nurse of each ward in the INH will attend these lectures to ensure that this leading nurse provide support to the staff during the trial. We will also encourage the nursing home physician to participate. Each staff member in the INH will be provided with the TIME manual, which describes the intervention step by step. They will also be given access to an educational film about TIME and a website to support the intervention. The three nurses who participated in the coeducation for the inclusion criteria in each unit in the INH will now hold the special responsibility for putting the model into practice based on the manual. These nurses will therefore receive three additional hours of education, training and role play related to the different components of TIME and the implementation of the intervention. In the trial, they are referred to as TIME administrators. Immediately after randomization and allocation, the project management team will contact the TIME administrators via telephone and instruct them to begin to implement the intervention according to the TIME manual for the patients included in the trial. This telephone call is made from a few days up to 1 week before the education and training sessions are given. The TIME manual is available online.

One specialist registered nurse from the education and training team will attend and supervise the TIME administrators’ first case conference on their first patient in their nursing home. For the remainder of the TIME intervention, and for the other patients included in the trial, the TIME administrators and the staff will carry out the intervention independently.

### Description of the TIME intervention

The actual assessment and treatment programme for individual patients is described in detail in the TIME manual, which provides a step-by-step guide to implementing the model. TIME consists of three overlapping phases: a registration and assessment phase; a guided reflection phase, including one or more case conferences; and an action and evaluation phase. These phases were adapted from and based on problem-solving methods in CBT [20] and are in line with reviews describing the “state of the art” for the management of NPS [4, 6]. The different components of TIME acting together thus provide an evidence-informed standardized approach to managing NPS.

In the registration and assessment phase, the nursing home physician performs an examination of the patient and the patient’s previous medical records and medications are critically reviewed. The staff gather personal background information, pain is assessed, behaviour and symptoms are registered in detailed 24-h daily records, and behaviour and symptoms are monitored with established clinical instruments, including the NPI-NH. This phase is described in detail in Table 1. The duration of this phase varies from one day up to 4 weeks, depending on the nature and burden of the symptoms. Following the registration and assessment stage, a guided reflection phase begins. In this stage, a case conference for the entire group of staff, including the physician, is conducted. Systematic reflection based on cognitive therapeutic principles is carried out. The goal of this guided reflection is to create a mutual understanding of the actual NPS of the patient and to tailor a detailed treatment plan that will be tested in the coming weeks. The case conference participants reflect on the situation using the cognitive problem-solving method, in which one problem is analysed at a time [20]. This reflection is performed systematically using a five-column sheet technique on a whiteboard, and the following five aspects are reviewed: assessed facts, interpretation, emotions, actions to take, and evaluation. The time frame and the agenda for the case conferences are outlined in Table 2. The last stage is the action and evaluation phase. In this phase, each treatment measure in the plan is put into action and is then systematically evaluated with the same assessment tools employed in the registration phase.

**Table 1:** The registration and assessment phase

Checklist for the registration and assessment phase of TIME			
Activity	Target symptoms:		
	Agree on the primary challenges for the patient using the Neuropsychiatric Inventory-Nursing Home Version (NPI-NH) to define precise target symptoms for the assessment		
	Observation of the target symptoms using a 24-h observation form	Staff	Responsible
	NPI-NH to assess other neuropsychiatric symptoms	Staff	
	^a^Cornell Scale of Depression in Dementia (CSDD) or another scale to assess possible symptoms of depression	Staff	
	Physical assessment	Nursing home physcian	
	Review of medication	Nursing home physcian	
	^b^Mobilisation-Observation-Behaviour-Intensity-Dementia Scale (MOBID-2) to assess possible pain	Staff Nursing home physcian	
	The Clinical Dementia Rating Scale (CDR) and/or the ^c^Mini-Mental State Examination (MMSE) to assess the dementia stage	Staff Nursing home physcian	
	^d^The Physical Self-Maintenance Scale (PSMS) to assess activities in daily life	Staff	
	Collection of resident life history, including preferences and resources, using an optional questionnaire	Staff interview the resident (if possible) and/or the next of kin	
	Make an appointment, i.e., set the date, time and place for the case conference	Staff/TIME administrator	

^a^Cornell Scale of Depression in Dementia (CSDD) [26, 27]

^b^Mobilisation-Observation-Behaviour-Intensity-Dementia Scale (MOBID-2) [45]

^c^Mini-Mental State Examination (MMSE) [46]

^d^Physical Self-Maintenance Scale (PSMS) [21]

**Table 2:** Agenda and time frame for the guided reflection meeting (case conference)

Agenda for guided reflection meeting (case conference) 1.5 h				
Activity	Preparation: Convene a meeting and prepare a meeting room with a blackboard or similar facilities (projector, if available). Check that a flip pad and markers are available	TIME administrators:		Responsible
		One is chairman for the meeting.		
		One takes notes on the whiteboard.		
		One writes the minutes on the 5-column sheet.		
	1. Status Report: Personal history and main points from the patient’s medical record are presented.	10–15 min	Decide in advance who should prepare and present the patient’s personal history and the main points from the medical record.	
	2. Create a problem list	10 min	Staff (as many as possible should attend the conference)	
	3. Prioritize problems from the list			
	4. Draw a 5−column sheet on the whiteboard: facts – interpretations (thoughts) - emotions – actions – evaluation	60 min	The leading registered nurse and the nursing home physician should attend the conference, if possible.	
	5. Describe facts from the registration and assessment phase: one problem at a time			
	6. Suggest interpretations – guided discovery – discuss and reflect on them			
	7. Describe any emotions experienced by the staff – with interpretations by the staff			
	8. Suggest ^a^SMART actions – based on the interpretations – decide how and when to perform an evaluation of the actions			
	9. Summarize interpretations and actions – close the meeting	5–10 min	TIME – administrator (chairman)	

^a^SMART (Specific-Measurable-Actual-Realistic-Time framed)

The time frame for the complete intervention with TIME will vary from 1 or 2 weeks up to 8 weeks depending on the severity and complexity of the NPS to be approached and the resources available in the nursing homes.

### Procedures for data collection

The patients’ demographic data, baseline data and primary and secondary outcomes will be collected by project nurses not affiliated with the nursing homes. All 10 assessors are nurses with substantial experience and formal training on the use of the assessment scales. They attended a one-day course on the use of the assessments scales before start of the trial. The assessments of all outcomes and covariates will be repeated at 8 and 12 weeks after baseline. The assessors will collect the data via telephone by interviewing staff members who know the patient best. The assessors will be blinded to the randomization of the nursing homes. The following data from patients’ medical records will be collected: age, gender, marital status, type of ward the patient lives in (regular somatic, special care units for dementia patients or other types), known diagnoses (chronic diseases), and dementia diagnosis.

The following data describing the nursing homes will be assessed by a questionnaire sent to the leading ward registered nurse at the start of the trial: the size of the nursing home (number of patients); the size of the unit and ward (the number of patients per unit and ward); the care factor (the number of nurses working per patient per work shift); the number of hours the nursing home physician works per patient per week in the nursing home/unit; and the number of employees per leading ward registered nurse.

Covariates that will be measured are: level of dementia, as assessed by the CDR; level of functioning in daily activities, as measured by the Physical Self-Maintenance Scale (PSMS; [21]; and physical health, as measured by the General Medical Health Rating Scale (GMHR; [22]. PSMS is a six-item scale that produces a sum score ranging from 6 to 30. A higher score denotes more severe functional impairment. GMHR is a one-item global rating scale with the categories good, fairly good, poor and very poor.

### Baseline data and primary and secondary outcome measures

A full description of the screening instruments used to assess the inclusion criteria and the primary and secondary outcomes is provided in Table 3. The primary outcome of the TIME trial is the difference in the change between intervention and control group in agitation/aggression at 8 weeks from baseline, as measured by the NPI-NH [17]. The Norwegian version of the NPI-NH has shown high inter-rater reliability and validity [23].

**Table 3:** Primary and secondary outcome measures

What is measured (scales/tools)	Characteristics and psychometric properties of scales/tools
Primary outcome measure: The difference between the intervention group and the control group in change from baseline at 8 weeks	
Agitation/aggression (single item from the NPI-NH)	Change from baseline of agitation and aggression, as defined by the Neuropsychiatric Inventory-Nursing Home version (NPI-NH) item agitation/aggression. The NPI-NH assesses the frequency (0–4) and the severity (0–3) of 12 psychiatric and behavioural symptoms. An item score is generated by multiplying frequency and severity (0–12). A higher score indicates more frequent and severe presence of NPS.
Secondary outcome measures: The difference between the intervention group and control group in change from baseline at 8 and 12 weeks	
Neuropsychiatric symptoms (NPI-NH)	12 items described in the Neuropsychiatric Inventory Nursing Home Version (NPI-NH). Range 0–12, as described above.
Subsyndrome of agitation (NPI-NH)	The NPI-NH subsyndrome agitation is defined as the sum of the scores of the agitation/aggression, irritability, and disinhibition items. Range 0–36.
Subsyndrome affective symptoms (NPI-NH)	The NPI-NH subsyndrome affective symptoms is defined as the sum of the scores of depression and anxiety items of the NPI-NH. Range 0–24.
Subsyndrome psychotic symptoms (NPI-NH)	The NPI-NH subsyndrome psychosis is defined as the sum of the hallucinations and delusions items. Range 0–24.
Neuropsychiatric symptoms (NPI-10 NH sum score)	The NPI-10 NH sum score is the sum of the first ten items in the NPI-NH, omitting the sleep disturbances and eating disorders (primarily vegetative symptoms) items. Range 0–120.
Caregiver occupational disruptiveness (NPI-NH)	In NPI-NH, the caregiver must rate how disruptive they consider each behaviour or symptom on a five-point scale. Range 0–5. A higher score indicates a more disruptive behaviour.
Agitation (CMAI)	The Cohen-Mansfield Agitation Inventory (CMAI), which measures 29 different types of agitation and the frequency at which they occur. Range for each item 1–7. Range total score 29–203. A higher score indicates more frequent agitation. Good validity and inter-rater reliability.
Depressive symptoms (Cornell)	The Cornell Scale for Depression in Dementia, which measures the frequency of symptoms of depression.
Quality of life (QUALID)	Quality of Life in Late-stage Dementia Scale, which measures 11 behaviours rated on a 5-point Likert scale. Range 11–55. A lower score indicates higher quality of life. Good validity and inter-rater reliability
Use and dosage of psychotropic and analgesic medication (defined as daily dosage (DDD))	Psychotropic and analgesic medication given both regularly and on demand. This will be assessed using a questionnaire and extracted from patients’ records. The assessment of the medication given on demand will be obtained from patients’ records at each visit and presented as the sum in mg used for the last 21 days. These drugs will be grouped according to the Anatomical Therapeutic Chemical Index.

The secondary outcomes include the difference in the change between the two groups in agitation/aggression at 12 weeks from baseline, as measured by the NPI-NH, in the change from baseline to 8 and 12 weeks in each of the other items of the NPI-NH, the NPI-NH 10 sum score, NPI-subsyndromal agitation score (aggression/agitation + disinhibition + irritability), NPI-subsyndromal psychosis score (delusion + hallucination) and affective symptoms (depression + anxiety). These subsyndromes are based on data from a previous principal component analysis [24]. The other secondary outcomes include the difference between the two groups in the change from baseline to 8 and 12 weeks in the following measures: agitation, as measured by the Cohen-Mansfield Agitation Inventory (CMAI; [25]; symptoms of depression, as measured by the Cornell Scale for Depression in Dementia (CSDD); [26, 27]; drug use and dosage of psychotropic and analgesic medications given both regularly and on demand, coded as defined daily dosage (DDD) and grouped according to the Anatomical Therapeutic Chemical index [28]; and quality of life, as measured by the Quality of Life in Late-stage Dementia Scale (QUALID); [23, 29].

### Qualitative and quantitative methods employed in the trial to answer research questions 2 and 3

#### Focus group interviews

Focus group interviews will be conducted after the intervention is completed. Four groups of five to eight caregivers and leaders of the INH will participate. One group of nursing home leaders, one group of TIME administrators and two groups of caregivers from the staff in different nursing homes will be arranged. Because of group dynamics, the questions posed can be discussed from several points of view. These dynamics can create new perspectives and views during the discussion. We will use an interview structure based on a semi-structured interview guide that asks informants to reflect on two main themes and follows up with open and exploratory questions posed by the interviewer [30]. These two themes are 1) the feasibility of the intervention, with an emphasis on the factors that promote or inhibit the implementation of TIME, and 2) the effects that the model has on learning and staff members’ experience of coping and mastery of strain in the face of the challenging behaviour of persons with dementia. If other key themes emerge spontaneously during the interviews, time will be allotted to develop these themes. Interviews will be transcribed. A qualitative content analysis will be used to explore the findings. Systematic text condensation [31] will be performed to provide a systematic description and to develop new concepts and understandings of the phenomena. Researchers will identify units in the text and then code and reorganize these units repeatedly to emphasize the meaning content of the data.

### Questionnaire surveys administered to the staff. Methods evaluating the extent and duration of the implementation of the model by use of the RE-AIM framework

A full description of the questionnaires, including respondents and the time point(s) at which they are administered, is provided in Table 4. The implementation of TIME will be followed and assessed from the start of the study to 1 year following the study based on the RE-AIM framework [32]. RE-AIM is a widely used system for following and evaluating interventions in organizations that aim to implement new methods of practice. RE-AIM is an acronym for Reach, Efficacy, Adoption, Implementation and Maintenance. In our trial, “Reach” refers to the proportion of the staff participating in the training, routine patient assessment, and subsequent conference meetings. This information will be recorded using a registration form to assess staff participation in education and training sessions and a self-developed questionnaire administered to the staff. “Efficacy” refers to each staff member’s intervention outcomes in terms of attitudes, mastery, knowledge and skills, which we will assess and analyse using data from the focus group interviews and questionnaires. The following questionnaires will be administered for this purpose: the General Nordic Questionnaire for Psychological and Social Factors at Work regarding mastery and social interaction (QPS-Nordic) [33], the Approaches to Dementia Questionnaire (ADQ) [34], and a brief self-developed questionnaire assessing perceived competence regarding NPS. Both QPS-Nordic and ADQ are validated questionnaires used to assess these domains.

**Table 4:** Questionnaires distributed to the staff and the leading ward registered nurse based on the RE-AIM-framework ^a^ for the evaluation of complex interventions

What is assessed	Questionnaire	Corresponding dimension of the RE-AIM framework	Time frame	Respondents in the nursing homes (NH)
Proportion of staff members participating in education and training sessions	A registration form to assess participation of staff in education and training sessions	Reach: proportion of staff in INH that actually participated in the intervention during the trial	At the start of the intervention during education sessions	All staff members in intervention nursing homes (INH)
Individual participation of staff members in effectuating the components of the model	Self-developed questionnaire	Reach: as above Maintenance: extent to which the model is sustained over time	6 and 12 months after the start of the intervention	All staff members in INH
Attitudes towards persons with dementia, mastery, social interaction, job satisfaction and self-assessment of competence with neuropsychiatric symptoms (NPS)	Approaches to the Dementia Questionnaire ^b^, QPS-Nordic ^c^ and a self-developed questionnaire for assessment of competence with NPS	Efficacy: outcomes regarding knowledge, skills and/or attitudes of the staff in NH	1 month before, and 6 and 12 months after the start of the intervention	All staff members in control nursing homes (CNH) and in INH
Clinical routines in place in NH, i.e., questions assessing daily routines of practice for assessment and treatment of NPS	Self-developed questionnaire based on evidence-informed best practice for assessment and treatment of NPS	Adoption: proportion of wards that will adopt the intervention Maintenance: extent to which the model is sustained over time	1 month before and 6 and 12 months after the start of the intervention	Leading ward registered nurse in INH and CNH
Fidelity to the main components in the model	Interview of TIME administrators by telephone using a checklist based on the components in the TIME manual	Implementation: extent to which the intervention is actually implemented	3 brief interviews, the first one 3 weeks after the start of the intervention and then at 1-month intervals	TIME administrators in INH
Organizational structure in the nursing homes: size of wards, type of unit, number of staff, etc.	Self-developed questionnaire	Implementation: possibility to assess and analyse implementation barriers and facilitators	At the start of the intervention	Leading ward registered nurse in INH and CNH

^a^RE-AIM framework: Reach-Efficacy-Adoption-Implementation-Maintenance [32]

^b^General Nordic Questionnaire for Psychological and Social Factors at Work (QPS-Nordic) [33]

^c^Approaches to Dementia Questionnaire (ADQ) [34]

In this type of intervention, “Adoption” refers to the proportion of wards and the percentage of the staff who actually adopt this method to manage NPS. We will use data from the focus group interviews and a self-developed questionnaire about participation in the routines of practice in the unit. “Implementation” refers to whether the intervention is carried out at the organization level as planned and with integrity. It will be assessed with a checklist once per month for 3 months after the start of the intervention. The checklist includes only the main components of the intervention derived from the checklist in the TIME manual. “Maintenance” refers to the degree to which the organization succeeds in maintaining the intervention after the project period. Maintenance will be measured with a self-developed questionnaire administered to units at 6 and 12 months after the intervention is implemented. This questionnaire will assess the extent to which the model and its components continue to be systematically applied. To answer the question of which factors inhibit or promote implementation, we will analyse the data from the focus group interviews and from the questionnaires.

### Data processing and statistical analysis of quantitative data

All data regarding the cluster randomized trial will be collected via telephone. The data will be registered online in the dataset prepared for the study using the data tool Checkbox via a web survey to the SQL database. The questionnaires used to collect all other data will be distributed to staff (regardless of job position) by mail and the results will be stored in the same manner as the trial data. At the 6- and 12-month assessments, questionnaires will be sent only to the staff who responded to the previous questionnaires.

The data will be presented as frequencies and percentages for categorical and means (standard deviations) for the continuous variables. The normality of continuous variables will be assessed graphically. If necessary, skewed data will be transformed. Differences in the changes in outcomes between the intervention group and the control group will be assessed by a linear mixed model with fixed effects for time component and group and the interaction between the two. A significant interaction will imply the differences in change between the groups. Random effects for patients nested within nursing homes and slopes (if significant) will be included into the model. Individual time point contrasts will be derived within each group at each time point with the corresponding 95 % confidence intervals and *p*-values. Linear mixed model correctly adjusts estimates for intra-cluster correlations as well as for intra-individual correlations due to repeated measurements in time. The model also handles unbalanced data by allowing inclusion of all available information, also from drop-outs.

### Trial status

The cluster randomized trial will be carried out from January to the end of June 2016.

Focus groups will be held in September and October 2016. The part of the trial evaluating the implementation process started in December 2015 and continue until the end of April 2017.

## Discussion

The main purpose of this trial is to improve the assessment and treatment of agitation in persons with dementia by examining the effect and implementation of the TIME intervention model. The strength of the model is that it was developed in nursing homes over a period of several years; thus, it takes into account the nursing home context. A pilot study showed its feasibility and further developed the model [13]. The model integrates pharmacological and nonpharmacological treatments for use in real-world implementation. The components of the model have a solid theoretical foundation [6, 20, 35]. Given that NPS often represent complex problems with multifactorial causes that interact with each other, often in unpredictable ways, multifaceted and complex interventions must be applied.

One of the challenges of psychosocial interventions is effectively and sustainably implementing them. Fixsen defined implementation as a specific set of activities combined in practice to introduce an activity or a programme with known components. Similar to an actual intervention (programme or model), implementation includes a set of activities and a set of outcomes [36]. Richards and Hallberg defined complex interventions as “Activities that include multiple components with the potential for interactions between them. When such an intervention is applied to the target population a number of possible and varied results are created” [37]. Based on this description, we claim that TIME satisfies the definition of a complex intervention [14]. An intervention’s complexity must also be considered based on the context—that is, the type of organization and the organization’s various participants [38]. A lack of an effect may reflect the failure of implementation rather than shortcomings of the implemented programme or model [38]. Therefore, the implementation of complex interventions is particularly demanding.

The Medical Research Council (MRC) defines an overarching framework for the development and evaluation of complex interventions. This recommendation was revised in 2008 to place greater emphasis on the importance of the process evaluation and adaptation to local contextual conditions compared with the previous recommendations [39]. In our trial, we will follow these recommendations and simultaneously apply an experimental design for measurements of effectiveness at the patient level and conduct an experimental evaluation of the implementation. Our reports on the TIME trial will follow the recommendations presented in the CONSORT 2010 statement: extension to cluster randomized trials [40].

A design that combines clinical effectiveness and implementation outcomes in one trial is called an effectiveness-implementation hybrid design [41, 42]. The main advantage of this hybrid design is that it can accelerate the translation of research findings into routine practice. It also allows the research team to evaluate the results regarding effectiveness in light of the degree of fidelity and adoption of the model. For this advantage to be realized, the implementation strategies in the trial cannot be overly complex. Thus the implementation strategies should not demand basic structural changes within the organization receiving the intervention. Although TIME is a complex intervention, we experienced during the pilot study that the intervention does not require significant changes within the organizations’ structures or routines, and the implementation costs were estimated to be low.

Our study design has some limitations. We do not require a precise diagnosis of dementia as an inclusion criterion; instead, we include patients with probable dementia, defined as a CDR score of one or higher. A previous study on a Norwegian NH showed that only approximately one-third to one-half of residents with dementia were assessed and given a diagnosis of dementia [1, 43]. In addition, several studies have shown that CDR staging based solely on an informant interview is a valid substitute for patient examinations [43, 44]. Therefore, even if a few patients included in our study do not fulfil all the criteria for a dementia diagnosis, the use of the CDR as a criterion for inclusion instead of a precise diagnosis of dementia will strengthen the external validity of our findings. Another limitation is the rather short follow-up time. The last visit in which the patient outcomes will be assessed is the 12-week visit, primarily due to resource limitations and to ensure staff compliance. To be considered clinically important, an intervention aimed at reducing NPS should show some measurable effects after 8 to 12 weeks. The data collection concerning the implementation process will nevertheless span a year to measure the sustainability of the intervention.

## Conclusion

The increasing percentage of the population with dementia will be a major challenge for health and care facilities in the coming years. Nearly all people who suffer from dementia experience NPS in the course of their disease. NPS like agitation, including physical or verbal aggression and excessive motor activity cause patients to experience profound suffering and a reduced quality of life and caregivers to suffer increased burden [4]. TIME is a multicomponent intervention based on the theoretical framework of CBT. The TIME trial is an effectiveness-implementation cluster randomized trial designed to assess both effects on NPS in persons with dementia residing in nursing homes and the implementation process at the staff and organization levels. An open pilot study conducted in 2010 showed that the intervention is feasible and found a reduction in patients’ agitation and mood symptoms and caregiver strain. The trial will take place in 30 nursing homes and will include 168 patients with dementia and a high degree of agitation. The aim of this project is to make an important contribution to improve the treatment of NPS. Furthermore, the project may result in an evidence-based model for assessment and treatment in both primary care and specialist care. The project will provide additional insight into how to sustainably implement complex interventions.

## Abbreviations

ADQ, approaches to dementia questionnaire; BPSD, behavioural and psychological symptoms in dementia; CBT, cognitive behavioural therapy; CDR, clinical dementia rating scale; CMAI, cohen-mansfield agitation inventory; CNH, control nursing homes; CSDD, cornell scale of depression in dementia; DDD, daily drug dosage; GMHR, general medical health rating scale; ICC, intracluster correlation coefficient; INH, intervention nursing homes; MOBID-2, mobilisation-observation-behaviour-intensity-dementia scale; NPI-NH, neuropsychiatric inventory-nursing home version; NPS, neuropsychiatric symptoms; SD, standard deviation; PSMS, physical self-maintenance scale; MMSE, mini-mental state examination; QUALID, quality of life in late-stage dementia; RE-AIM, reach-effectiveness-adoption-implementation-maintenance; QPS, general nordic questionnaire for psychological and social factors at work; SMART, specific-measurable-actual-realistic-time; SQL, structured query language; SPSS, statistical product and service solution; TIME, targeted interdisciplinary model for evaluation and treatment of neuropsychiatric symptoms.
